# Efficacy of postural techniques assessed by videofluoroscopy for myasthenia gravis with dysphagia as the presenting symptom: a case report

**DOI:** 10.1186/1752-1947-4-370

**Published:** 2010-11-19

**Authors:** Hui-Chun Juan, Isabel Tou, Shu-Chen Lo, I-Hsien Wu

**Affiliations:** 1 Department of Physical Medicine and Rehabilitation, Chi Mei Medical Center, Liouying, Taiwan; 2 Department of Physical Medicine and Rehabilitation, Chi Mei Medical Center, Tainan, Taiwan

## Abstract

**Introduction:**

Oropharyngeal weakness leading to dysphagia is rarely the presenting symptom of myasthenia gravis, but it can be a significant source of morbidity and mortality. The earliest possible diagnosis of myasthenia gravis should be made for better management of this cause of treatable dysphagia. A detailed evaluation of swallowing by videofluoroscopy can assist in making an accurate diagnosis and in individualizing appropriate diet compensatory techniques.

**Case presentation:**

We present the case of a 57-year-old Taiwanese man with dysphagia as the presenting symptom of myasthenia gravis, and evaluate the pathological findings of swallowing and effectiveness of compensatory postural techniques for dysphagia using videofluoroscopy.

**Conclusions:**

Videofluoroscopy is a valuable technique for evaluating myasthenia gravis dysphagia, because it allows swallowing interventions to be precisely individualized in accordance with the results obtained.

## Introduction

Myasthenia gravis (MG) is an autoimmune disorder in which autoantibodies are directed against acetylcholine receptors in the neuromuscular junctions [1]. It is characterized by painless and fatigable weakness of skeletal muscles. Although over 60% of patients with MG have ocular symptoms at presentation, dysphagia related to weakness of the oropharyngeal muscles can also be a presenting symptom. Dysphagia with aspiration is a significant source of morbidity and mortality in MG [2]; consequently, a detailed assessment of swallowing is very important in patients with MG dysphagia.

Videofluoroscopy is a useful evaluation tool for observing the oral, pharyngeal, and esophageal stages of swallowing physiology in patients ingesting radiopaque foods [3], and swallowing evaluation by videofluoroscopy in patients with MG has been widely utilized in previous studies. However, subsequent videofluoroscopic monitoring of compensatory postural techniques to guide appropriate management of swallowing in MG has been less frequently mentioned. Here, we report the case of a patient with dysphagia as his main presenting symptom and describe the use of videofluoroscopy to evaluate his swallowing status and the effectiveness of compensatory postural techniques.

## Case presentation

A 57-year-old Taiwanese man presented to our facility with a one-month history of progressive difficulty in swallowing, particularly liquids. In addition, a body weight loss of 10 kg was noted. His medical history included chronic sinusitis and chronic serous otitis media. A chest X-ray revealed incidental right middle lobe collapse when he was admitted for surgical treatment for chronic paranasal sinusitis. Obstructive pneumonitis with a right lower lung field mass lesion was suspected. A physical examination revealed left ptosis, dysarthria, and mild bilateral shoulder girdle weakness without a definite diurnal change. He was subsequently diagnosed as having MG based upon the decremental response to repetitive stimulation on electrophysiological testing in association with a positive anti-acetylcholine receptor antibody test. The MG stage was assessed as grade IIA according to the Osserman classification at that time.

**Figure 1:**
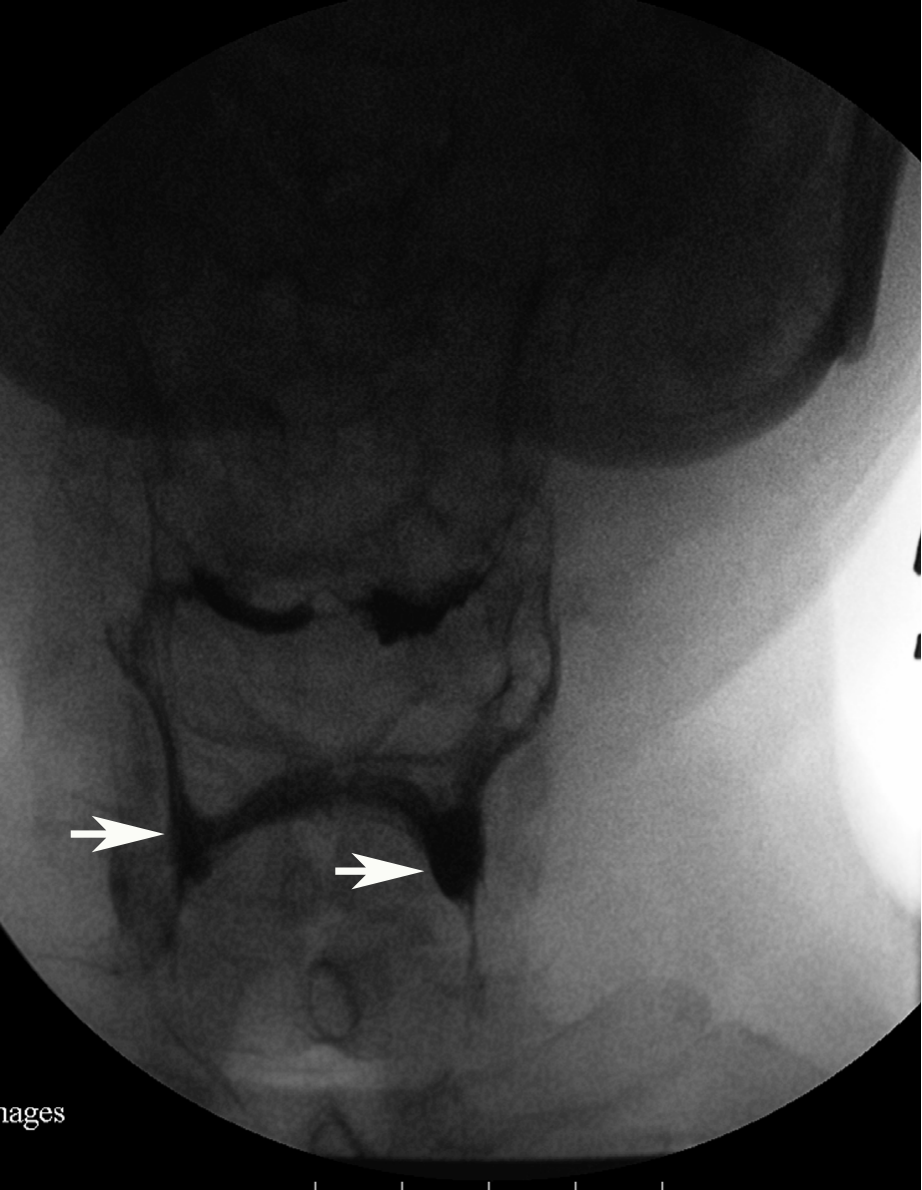
**Videofluoroscopic image, anteroposterior projection, first swallow with pudding**. The image shows small volume residues in the valleculae and the pyriform sinuses (bilateral pyriform sinuses indicated with white arrows).

**Figure 2:**
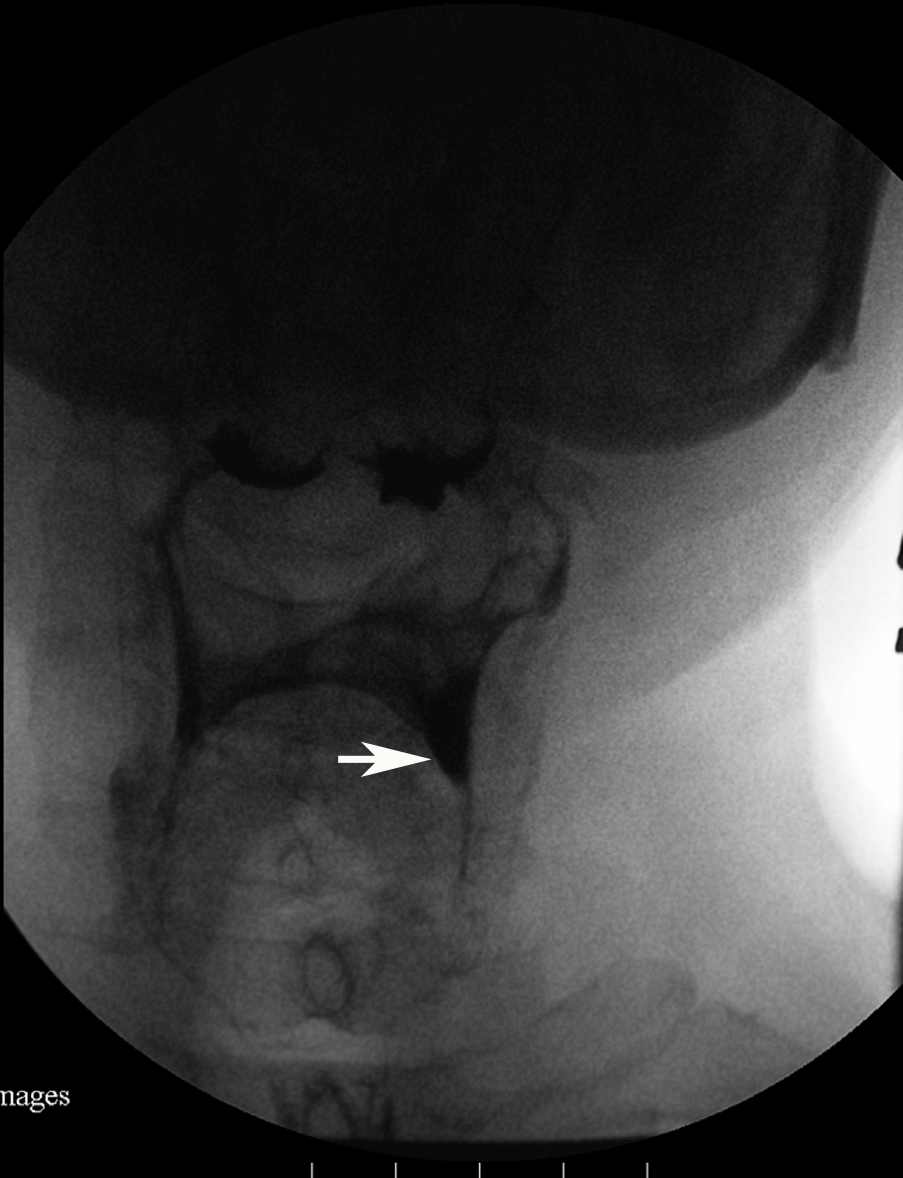
**Videofluoroscopic image, anteroposterior projection, after five swallows with pudding**. Residues were increased in volume, particularly in the left pyriform sinuses (indicated with white arrow), compared with Figure 1.

**Figure 3:**
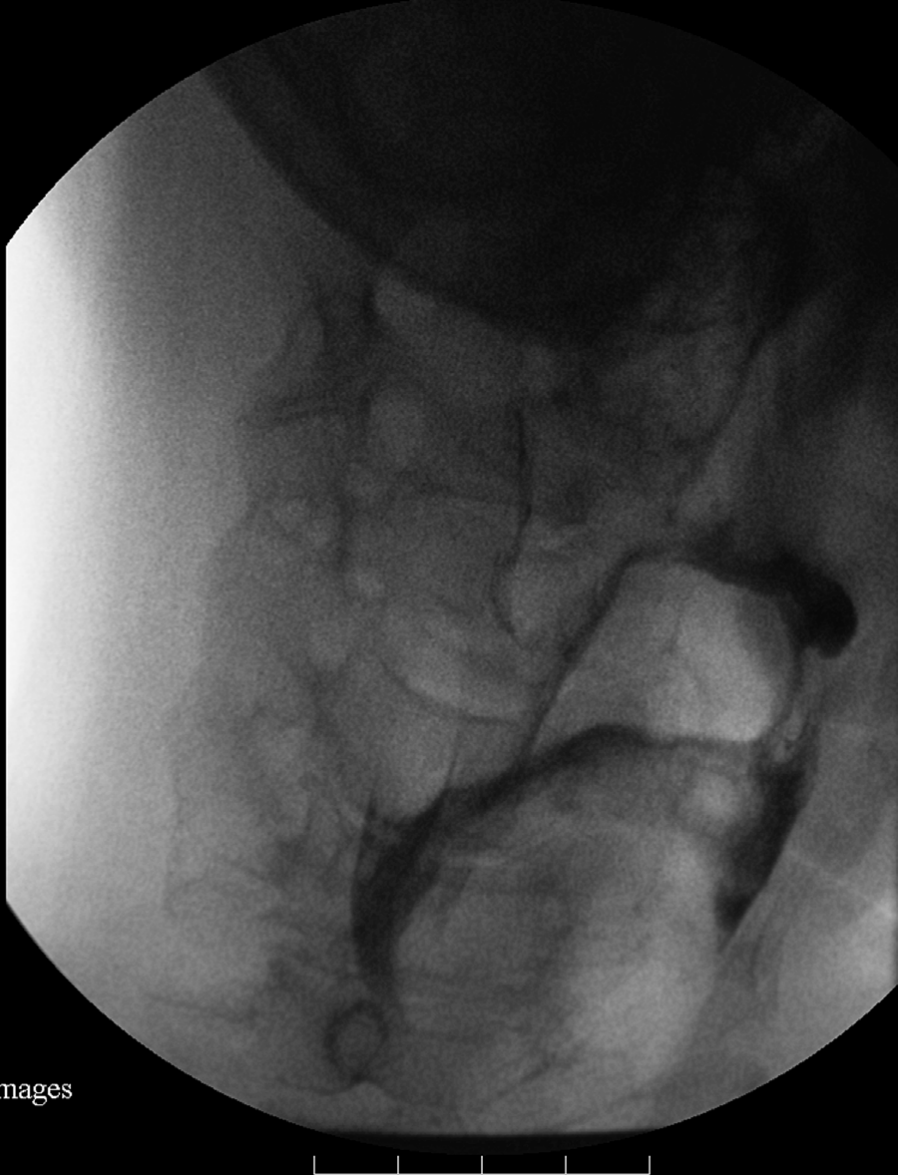
**Videofluoroscopic image, anteroposterior projection, with the head rotated to the left side**. Residues decreased in volume and no aspiration was detected.

A clinical swallowing evaluation by a speech/language pathologist showed our patient had mild difficulty in oral preparation and transport during trial swallows of food and liquid. A delay in his swallow reflex trigger without fatigability was also noted. The strength of his oral muscles, head rotators, extensors and flexors were assessed as grade four by manual muscle testing. We accordingly conducted a videofluoroscopic study with three mL boluses of thin liquid, thick liquid, and pudding administration. In the oral phase of swallowing, it was noted our patient had poor oral holding and tongue movement. During the pharyngeal phase, we observed our patient had a delayed swallowing reflex (onset of swallow reflex trigger: 2.5 seconds) with incomplete laryngeal closure, and poor pharyngeal wall motility. Silent aspiration was observed on administration of three mL pudding. Residual food was pooled in the bilateral pyriform sinuses and valleculae after swallowing three consistencies of sample material. After repeated consecutive swallows, the residues in the bilateral pyriform sinuses increased in volume, particularly on the left side (Figure 1 and 2). However, the residues decreased in volume when his head was turned to the left side and there was no evidence of silent aspiration (Figure 3). In contrast, chin tuck or head tilting to the right side were ineffective measures for reducing the pharyngeal stasis. Our patient was instructed on the correct postural technique to adopt (head turning to the left side) in order to improve his swallowing safety and efficiency. Due to MG crisis-related respiratory failure, our patient was admitted to an intensive care unit and underwent thymomectomy and tracheostomy. After seven plasma exchanges were performed, his condition improved and his tracheostomy tube was successfully removed. His swallowing ability was evaluated again after he was transferred to an ordinary ward. Mildly delayed swallow reflex (onset of swallow reflex trigger: 1.5 seconds) and occasional choking were noted only when he consumed about 10 mL of thin liquid from a cup. Following his discharge, he was able to eat food of ordinary consistency.

## Discussion

Fatigable muscle weakness is characteristic of MG and weakness of the oropharyngeal muscles can produce dysphagia, which is a frequent symptom in MG [4]. During the course of MG, at least 40% of patients suffer from dysphagia [4]. Dysphagia can be the presenting symptom in 6% to 15% of patients with MG [5], but it is rarely the sole manifestation. Dysphagia can also recur or worsen in patients with chronic MG, and it may signal exacerbation of the disease or other unusual causes (for example, cricopharyngeal sphincter achalasia) [6]. In order to distinguish dysphagia due to MG from other neurological disorders, the characteristics of dysphagia in MG have been discussed in previous studies. Disturbance in the pharyngeal phase is the most frequent swallowing abnormality in patients with mild to moderate MG [7]. The most common pathological finding in the pharyngeal phase is delayed onset of laryngeal elevation and epiglottic inversion [7], which was also noted in our patient, and this can lead to a risk of aspiration. Patients with oral phase abnormality, which is relatively mild and less frequent, generally present with lip spill, extended and incomplete chewing, and difficulties forming a cohesive bolus with the tongue. Further study is needed to evaluate the characteristics of patients with MG of different severities. In our patient, his swallowing ability improved significantly secondary to surgical and medical treatment. However, improvement of dysphagia in response to medical therapy for myasthenia gravis is variable and often less satisfactory than the response of other manifestations, according to previous studies [8]. Further monitoring of MG-related dysphagia could be necessary after other symptoms improve.

For an evaluation of dysphagia, a thorough history and clinical examination provide valuable information. The swallowing ability of patients with myasthenia with dysphagia typically, but not always, shows fatigability during meals and as the day progresses. However, clinical examination alone is insufficient to detect and grade dysphagia in MG, and additional instrumental assessment tools may be necessary [9]. According to the study of Colton-Hudson *et al*. in 2002, the severity of MG dysphagia as determined by videofluoroscopic study was worse than that predicted by clinical evaluation. They accordingly suggested routine videofluoroscopic examination [7]. Videofluoroscopy is regarded as the gold standard in dysphagia diagnosis and management [3]. For patients with MG with dysphagia as the presenting or sole manifestation, videofluoroscopy is helpful for early and accurate diagnosis because insidious fatigability after consecutive swallowing can be detected, as in our patient. Videofluoroscopy can also be combined with the Tensilon test to assist in diagnosis of bulbar MG. The combination is particularly valuable for the subgroup of patients with MG who have prominent bulbar symptomatology, and it is more reliable than videofluoroscopy alone [10]. After medical management of MG, videofluoroscopy is indicated for following up the course of dysphagia to modify subsequent treatment strategies, particularly for those patients with dysphagia that does not improve as quickly as other manifestations. The limitations of videofluoroscopy swallow studies are generally related to radiation exposure; however, radiation exposure is rarely a limiting factor in adults [3]. Fiberoptic endoscopic evaluation of swallowing (FEES) is another common instrumental swallowing evaluation tool, which has greater portability than videofluoroscopy and involves no radiation exposure [3]. For individuals with physical limitations that prevent the use of fluoroscopy (for example, those who cannot be transported to the radiological ward or those who are unable to sit in an upright position), FEES can be beneficial. FEES is also more useful than videofluoroscopy for direct visualization of the anatomy of pharynx, larynx and vocal cord. Although there are some widely applied instrumental tools for evaluation of dysphagia, we chose videofluoroscopy in our patient's case because it can analyze functional impairment of swallowing mechanisms and test the efficacy of compensatory diet modifications, postures and behavior techniques.

In the management of MG, dysphagia is an important symptom to consider. In addition to medical management, swallowing therapy also plays an important role. Appropriate interventions include diet modification, behavioral techniques, postural techniques, and, if necessary, non-oral routes of feeding. Active exercises to maximize the strength of the oropharyngeal muscles are generally limited by fatigability and not recommended for dysphagia associated with MG [7]. Diet modification, postural techniques and behavioral techniques aim to improve swallowing safety and efficiency while allowing for oral feeding. Behavioral techniques include some compensatory swallowing skills (for example, effortful swallow, Mendelsohn maneuver, supersupraglottic swallow) to reduce aspiration and/or improve pharyngeal clearance. Postural techniques include compensatory postures, and there are some indications for their use. However, none of these techniques are effective for all patients. For example, head rotating to the weak side diverts the bolus to the contralateral stronger side and it is appropriate for unilateral pharyngeal weakness. Oral and pharyngeal weakness is an indication for head tilting toward the stronger side. When reduced oral bolus control with aspiration before or during the swallow is detected, the chin-tuck maneuver maybe helpful [3]. However, for our patient, only a head-tilting posture was effective according to videofluoroscopic assessment. Therefore, precise clinical and instrumental evaluations are necessary in selecting the most appropriate technique(s) to use. Our patient safely maintained an oral diet before his MG crisis using compensatory postural techniques, and it indeed improved his quality of life.

## Conclusions

For patients with MG with dysphagia as the presenting symptom, videofluoroscopy is helpful for diagnostic differentiation and swallowing therapy is an important intervention. On the basis of our patient's case, we can conclude that postural techniques are effective for patients with MG dysphagia. Proper postural techniques can maintain adequate oral nutrition and hydration of such patients while minimizing the risk of aspiration, and this is significant for improving quality of life. The efficacy of these techniques should be demonstrated by videofluoroscopic survey. We accordingly suggest routine videofluoroscopic evaluation for dysphagia related to MG to assist diagnosis and aid in preparing an individualized plan for swallowing therapy.

## Consent

Written informed consent was obtained from the patient for publication of this case report and any accompanying images. A copy of the written consent is available for review by the Editor-in-Chief of this journal.

## Competing interests

The authors declare that they have no competing interests.

## Authors' contributions

HCJ is the principal author who performed the literature search and drafted the case report. IT consulted with our patient and had input into the discussion. SCL performed the videofluoroscopy and swallowing evaluation. IHW reviewed the literature and defined the content of discussion. All authors read and approved the final manuscript
